# Insights into volatile fatty acid production and fermentation pathways of microalgae biomass combined with vinasse and glycerol

**DOI:** 10.1007/s11356-026-37738-4

**Published:** 2026-04-11

**Authors:** Dicla Cesário Pereira de Oliveira, Francyelle Karolynne Vieira Machado, Agnes Adam Duarte Pinheiro, Federico Battista, Mario Takayuki Kato, Lourdinha Florencio, Wanderli Rogério Moreira Leite

**Affiliations:** 1 Department of Civil and Environmental Engineering, Laboratory of Environmental Sanitation. Cidade Universitária, Federal University of Pernambuco, Recife, 50670901 Brazil; 2 Department of Biotechnology, Laboratory of Chemical Engineering for the Environment and Bioprocesses. Via Strada Le Grazie 15, University of Verona, 37134 Verona, Italy

**Keywords:** Dark fermentation, Acidogenesis, Cosubstrate, Hydraulic retention time, 1,3 propanediol, Metabolic pathways

## Abstract

The fermentation of industrial wastes derived from water recovery (pretreated microalgae biomass, PTMB), biodiesel (glycerol, GLY), and ethanol (vinasse, VIN)) is a promising sustainable alternative for producing volatile fatty acids (VFA). In this study, controlled fermentation experiments were performed using PTMB-VIN and PTMB-GLY mixtures with varying substrate concentrations and retention times to optimize the VFA yield and production efficiency. Acid fermentation was optimized at a 3-day hydraulic retention time, and methanogenic reactions were enhanced at longer hydraulic retention times. Increasing the organic loading rate resulted in a high VFA yield (13.10 g _VFA-COD_. L^−1^) and a conversion rate of approximately 40%. GLY fermentation followed the oxidative and reductive pathways at a balanced redox potential. PTMB is linked to the oxidative pathway, and GLY is involved in the reductive pathway. Tests with PTMB-GLY resulted in limiting conditions, but significant observations regarding the metabolic pathway and the effect of PTMB were made.

## Introduction

High population growth combined with the traditional linear economy involving raw material extraction, manufacturing of products, and disposal of generated waste is prevalent in numerous countries, which has led to increasing waste and the depletion of natural resources. The World Bank estimates that 3.4 billion tonnes of waste will be generated globally each year by 2050. The transition to a circular bioeconomy is being implemented as a waste management tool because it promotes the reuse, reprocessing, and recovery of value-added products, waste treatment, reduces pollution, and lowers the dependence on nonrenewable resources (Lü et al. 2021; Dahiya et al. 2023).

Dark fermentation (DF) is an anaerobic microbial process that converts simple carbohydrates or glycerol into short-chain organic acids, hydrogen, and carbon dioxide, with volatile fatty acids (VFA) as the main acidogenic byproducts (Guo et al. 2010). Short hydraulic retention times (HRT) can suppress methanogenesis and favor acidogenesis (Lü et al. 2021); however, HRT reduction alone is insufficient for VFA accumulation. Zhou et al. (2018) and Peces et al. (2021) evaluated stepwise reductions in HRT from 15 to 2 d at a constant organic loading rate (OLR) of 1 g chemical oxygen demand (COD) L⁻^1^ d⁻^1^ and showed that VFA accumulation depends on the combined management of HRT, OLR, pH, and substrate type. Similarly, Llamas et al. (2022) reported that a gradual HRT reduction from 10 to 2 d during microalgal fermentation enhanced microbial adaptation, achieving 32% VFA_COD_/COD_in_ at an HRT of 4 d. Overall, these studies indicate that stable VFA production in DF systems results from the interaction between operational parameters and substrate complexity, rather than from HRT control alone.

The VFA produced by the DF of organic waste streams are attracting increasing interest because of their growing market demand, sustainability, and ecofriendly attributes. They are used in the food, pharmaceutical, and cosmetic industries, and for the biosynthesis of polyhydroxyalkanoate (PHA), bioplastics, and biohydrogen (Frison et al. 2015; Strazzera et al. 2021; Llamas et al. 2022). This is an alternative strategy for the synthesis of VFA from petrochemical compounds.

Studies suggest using a mixture of substrates as a strategy to improve anaerobic digestion (AD), as it allows the combined treatment of more than one organic waste, promoting benefits such as the dilution of toxic compounds, nutrient regulation (C/N ratio), synergy between different microorganisms, and increased yields of their target products (Silva et al. 2024). Using wastes such as microalgae (MB), vinasse (VIN), and glycerol (GLY) has been studied (Takeda et al. 2022; Eng et al. 2022; Pinheiro et al. 2024).

Microalgae are mainly composed of proteins, lipids, and carbohydrates and can be obtained as waste from wastewater treatment plants in high-rate lagoons. However, the low C/N ratio and composition of their cell wall, comprising proteins, cellulose, hemicellulose, and carbohydrates, promote their recalcitrance as enzymatic reactions are hindered, reducing the potential to produce VFA, biogas, and other value-added products. Improving hydrolysis to increase the availability of soluble compounds to microorganisms is one method of addressing these issues (Wang et al. 2022; Silva et al. 2024).

Brazil produced approximately 28 billion liters of ethanol during the 2023/2024 harvest season, accounting for nearly 27% of global production (RFA 2024). Sugarcane vinasse (VIN), generated from 8‒15 L per liter of ethanol, is commonly reused for fertirrigation; however, its high organic content highlights its potential for energy recovery. -VIN contains organic acids, sugars, alcohols, aldehydes, ketones, and esters, which support anaerobic fermentation (ANP 2023; Parsaee et al. 2019). Brazil ranks fourth globally in biodiesel production, generating approximately 1.0 kg of waste GLY per 10 kg of biodiesel (Sunarno et al. 2020). GLY is a low-value, readily biodegradable by-product that has been widely reported as an effective carbon source for anaerobic fermentation, supporting VFA, hydrogen, and ethanol production (Silva et al. 2020; Chilakamarry et al. 2021; Wang et al. 2022). Takeda et al. (2022) showed that VIN–GLY co-digestion increased volumetric methane production by 12%, with COD removal above 80%, indicating high substrate biodegradability and the role of GLY as a readily fermentable carbon source. Similarly, Silva et al. (2024) reported higher methane yields during VIN–GLY co-digestion than VIN mono-digestion (655 ± 35 NmL CH₄ g⁻^1^ VS).

Despite the current maturity of biotechnological approaches using carboxylate platforms, some important aspects of bioreactor operation remain unclear, such as the appropriate HRT and OLR, particularly when DF is applied to pretreated microalgae biomass (PTMB) mixed with VIN or GLY using semi-continuous feed tank reactors. Thus, two key questions arose in the present study: 1) How do different carbon sources (PTMB, VIN, and GLY) interact in the VFA fermentation reactor? 2) What HRT and OLR values support higher VFA bioconversion stability? Addressing these questions may improve the DF process and advance the eco-efficiency of biorefineries.

## Materials and methods

### Anaerobic microorganisms for inoculation and substrates

The original mixed culture was obtained by self-inoculation of VIN and microalgae. This microbial consortium was maintained active through consecutive mixed microalgae biomass and VIN feeding under mesophilic conditions (30 ± 2 °C) and agitated at 120 RPM approximately 200 days prior to the beginning of the study (Pinheiro et al. 2024).

In this study MB, sugarcane VIN, and biodiesel residual GLY were used. MB was sourced from a high-rate pond used to polish the effluent of an anaerobic upflow sludge blanket reactor (UASB) installed in Recife (Northern Brazil). Sampling was conducted using a 0.250 mm stainless-steel sieve to improve the biomass concentration. Optical microscopy (Leica-DME, Leica, Wetzlar, Germany) revealed a predominance of the genera *Desmodesmus*, *Closterium*, and *Coelastrum*. The MB was subjected to hydrothermal autoclaving pretreatment at 120 °C at 1.0 kgf.cm^−2^ (Passos and Ferrer 2015); therefore, only the PTMB was used as a substrate.

VIN was collected from a sugarcane ethanol plant in Vitória de Santo Antão, Pernambuco, Brazil. Crude GLY was supplied by a pilot-scale biodiesel plant at the Experimental Biorefinery for Solid Organic Waste of the Federal University of Pernambuco. It was then purified by acidification and phase separation to remove waste oils (Hu et al. 2012). Consequently, the GLY content increased from 40 to 55% (w/w), and this phase, namely the residual GLY, was used to feed the biological system.

Substrates were analyzed upon collection and then stored at 4 °C. The main organic and inorganic characteristics of the substrates are listed in Table 1. The protein, carbohydrate, lipid, and metal characteristics were obtained from Silva et al. (Silva et al. 2024). The pH of the feed was adjusted using 40% NaOH and 1 M HCl.

**Table 1:** Organic and inorganic compounds present in microalgae biomass (MB), pretreated MB (PTMB), vinasse (VIN) and residual glycerol (GLY) (mean ± standard deviation)

Variables^1^	MB	PTMB	VIN	GLY
pH	7.3 ± 0.3	7.6 ± 0.1	3.7 ± 0	3.0
COD (g O_2_.L^−1^)	46 ± 13.5	52 ± 23.1	27 ± 2.5	1231 ± 4.1
SCOD (g O_2_.L^−1^)	3.4 ± 3.5	11.1 ± 6.1	15.7 ± 1	992 ± 10.8
TKN (mg.L^−1^)	1380 ± 454	1138 ± 214	239 ± 123	n.d.^2^
C/N ratio	14.2	21.5	48.8	n.a.^3^
TS (g.L^−1^)	33.4 ± 27.4	26.7 ± 3.6	57.6 ± 34.3	7.70 ± 1.2
TVS (g.L^−1^)	27.1 ± 22.8	19.4 ± 2.4	10.3 ± 4.2	2.90
Proteins (% TS)	29.7	n.a	n.a	n.a
Lipids (% TS)	18.1	n.a	n.a	n.a
Carbohydrates (% TS)	15.3	n.a	n.a	n.a
Al (mg.L^−1^)	52 ± 1	n.a	69 ± 2	n.d
P (mg.L^−1^)	117 ± 6	n.a	39 ± 3	6.8 ± 0.7
S (mg.L^−1^)	119 ± 3	n.a	239 ± 21	5.2 ± 1
Zn (mg.L^−1^)	3.0 ± 0.1	n.a	0.7 ± 0.1	0.2 ± 0.3
Fe (mg.L^−1^)	3.1 ± 0.6	n.a	36 ± 2	9.5 ± 0.8
K (mg.L^−1^)	156 ± 3	n.a	1571 ± 55	48 ± 3
Ca (mg.L^−1^)	1615 ± 93	n.a	390 ± 15	6 ± 3
Na (mg.L^−1^)	100 ± 2	n.a	37 ± 1	31198 ± 1621
Mg (mg.L^−1^)	470 ± 9	n.a	179 ± 9	0.8 ± 0.1
Mn (mg.L^−1^)	16 ± 1.01	n.a	2.9 ± 0.03	0.43 ± 0.03
Cu (mg.L^−1^)	0.40 ± 0.01	n.a	0.10 ± 0.01	1.10 ± 0.03
Mo (mg.L^−1^)	0.01 ± 0.01	n.a	0.01 ± 0.01	0.07 ± 0.04
Pb (mg.L^−1^)	0.13 ± 0.02	n.a	0.01 ± 0.01	0.02 ± 0.02
Cr (mg.L^−1^)	0.11 ± 0.01	n.a	0.06 ± 0.01	n.d
Ni (mg.L^−1^)	0.09 ± 0.01	n.a	0.01 ± 0.01	n.d
V (mg.L^−1^)	0.08 ± 0.01	n.a	0.05 ± 0.01	n.d
Co (mg.L^−1^)	0.04 ± 0.01	n.a	0.01 ± 0.01	n.d
Cd (mg.L^−1^)	0.004 ± 0.001	n.a	n.d	n.d

^1^COD: chemical oxygen demand; SCOD: soluble COD; TKN: total Kjeldahl nitrogen; C/N: carbon to nitrogen ratio expressed as COD/TKN; TS: total solids; TVS: total volatile solids

^2^n.d.: not detected, below detection limits (in mg.L^−1^): Cr: 0.001; Ni: 0.005; V: 0.001; Co: 0.002; Cd: 0.001

^3^n.a.: not analyzed

### Experimental setup

DF tests were performed in an 1.8 L working volume acidogenic reactor (AR) designed in an acrylic, cylindrical, 13 × 25 cm in size (diameter × height), with 20% headspace for the gas phase. It was equipped with a magnetic stirrer (150 rpm) and kept in a 30 °C temperature-controlled room.

The tests lasted for 340 d and were divided into six phases: I-A, I-B, I-C, I-D, II-A, and II-B. The AR was fed with a mixture of PTMB and VIN in phase I. Next, a mixture of PTMB and GLY was used as the substrate for phases II-A and II-B. The binary mixtures comprised 50:50% COD for phases I-A, I-B, I-C, and I-D (PTMB-VIN) and 70:30% COD for phases II-A and II-B (PTMB-GLY). The main feed characteristics are listed in Table 2. The pH was adjusted to 5.0‒6.0 with 40% NaOH in all phases.

**Table 2:** Main characteristics of the feed substrate used to feed the AR during the phases (mean ± standard deviation)

	Phase I (PTMB + VIN)				Phase II (PTMB + GLY)	
Variables	I-A	I-B	I-C	I-D	II-A	II-B
pH	5.5 ± 0.1	5.5 ± 0.1	6.0 ± 0.1	5.0 ± 0.1	5.0 ± 0.1	5.0 ± 0.1
COD (g.L^−1^)	20.8 ± 3.0	20.1 ± 4.4	40.7 ± 12.6	22.2 ± 3.9	29.6 ± 18.8	37.6 ± 10.2
SCOD (g.L^−1^)	11.3 ± 2.0	11.3 ± 2.1	16.4 ± 2.1	10.1 ± 1.8	15.9 ± 0.1	15.1 ± 5.4
TKN (mg.L^−1^)	1089 ± 195	256 ± 47	136 ± 17	987 ± 182	187 ± 2	754 ± 273
C/N	8.1	33.4	126.8	9.6	67	21.2
TS (g.L^−1^)	17.85	16.9 ± 0.65	38.8 ± 16.47	15.98 ± 1.63	22.44 ± 0.43	19.44 ± 4.79
TVS (g.L^−1^)	12.68	12.69 ± 0.43	29.73 ± 12.79	12.29 ± 1.34	17.33 ± 0.32	15.3 ± 4.36

The substrate was characterized by a controlled low pH (5.0–6.0), high organic solids content (> 70% TVS), and COD ranging from approximately 20‒40 g L⁻^1^. The soluble fraction represented 40–56% of the total COD, indicating that the substrate was partially readily available for anaerobic microorganisms. Variations in total Kjeldahl nitrogen (TKN) among the experimental phases were mainly attributed to PTMB, which was sourced from a high-rate pond used for nutrient removal from wastewater streams (Table 1).

Variations in the influent COD under Phase I conditions are a sign of the intrinsic complexity of the real substrates. For instance, PTMB contributed 50% of the influent COD in Phase I and exhibited high COD variability (52 ± 23.1 g O₂ L⁻^1^; Table 1), whereas VIN showed comparatively stable COD values. To ensure experimental comparability, feed volumes were adjusted to maintain a constant OLR based on the COD within each experimental phase. HRT was varied as an operational parameter under investigation (Table 3). All conditions were operated for at least eight hydraulic retention times to ensure steady-state behavior. During phases I-D and II-A, the reactor headspace was briefly opened while feeding owing to inlet tube issues; the operation proceeded under airtight conditions afterwards.

**Table 3:** Operational setup tested in 6 phases considering different substrate mixtures, applied OLR and HRT

Period (days)	Elapsed time (days)	Phase	Mix of substrates	Theoretical OLR (gCOD.L^−1^.d^−1^)	HRT (d)
01–38	37	I-A	PTMB-VIN	5.62	4
39–107	68	I-B	PTMB-VIN	5.62	3
108–171	63	I-C	PTMB-VIN	11.25	3
172–244	72	I-D	PTMB-VIN	11.25	2
245–279	34	II-A	PTMB-GLY	11.25	2
280–346	66	II-B	PTMB-GLY	11.25	4

### Analytical methods

The AR-fermented effluent was checked twice per week for TS, TVS, total ammonia nitrogen (TAN), COD, soluble chemical oxygen demand (SCOD), total alkalinity (TA), and partial alkalinity (PA) using standard methods (Awwa et al. 2017). The redox potential (ORP) and pH were measured using a portable multi-probe meter (HQ40d; Hach Instruments, Loveland, CO, USA). The C/N ratio was calculated using Eq. (1), which considers the carbon fraction (C) as the total organic carbon, COD, and TKN:

1 $$C/N ratio= \frac{0.425\times COD\times 1000-2.064}{TKN}$$

Total and individual VFAs, including acetic (HAc), propionic (HProp), butyric (HBut), pentanoic (HPent) and caproic (HCap) acids, were evaluated in the AR effluent using gas chromatography (GC) coupled to a flame ionization detector (300 °C) (Agilent Technologies 7890 A, J&W GC column DB-WAXERT 122–7332; 260 °C; 30 m 250 µm × 0.25 µm, hydrogen as carrier gas; Agilent Technologies, Santa Clara, CA, USA). During the analysis, the temperature started at 80 °C and reached 200 °C. The yield of VFA production (Y_VFA_) was estimated using Eq. (2), which considers the VFA concentration expressed as COD (VFA-COD), HRT, and mass of COD fed daily into the AR (mCOD_inlet_) taken from the OLR values in Table 2.

2 $${Y}_{VFA}(\%)=\frac{VFA(\frac{{g}_{VFA-COD}}{L})}{HRT (day)}\times \frac{1}{{mCOD}_{inlet}(\frac{g}{L\times day})}\times 100$$

Biogas production was measured using a 3.0% NaOH solution. Gas samples were collected from the AR headspace in gasbags and analyzed for methane (CH_4_), carbon dioxide (CO_2_), hydrogen, nitrogen (N_2_) and oxygen (O_2_) using GC coupled to a thermal conductivity detector (200 °C) and a capillary column (Agilent Technologies GC 7890 A, carboxen column 1010 PLOT, 30 m × 0.53 mm using air as the gas carrier).

#### Fermentation pathways of glycerin and microalgae (Phase II-B)

Metabolic pathways were studied in phase II-B to determine whether oxidative or reductive pathways were involved. Thus, alcohols (ethanol, glycerol, and 1,3 propanediol), total sugars (sum of saccharose, glucose, and fructose), other acids (succinic and lactic acids), and pyruvate were analyzed in the fermented samples collected in this phase. Alcohols and sugars were measured by high-performance liquid chromatography coupled to a refractive index detector (Shimadzu LC-20AT, (Shimadzu Corporation, Kyoto, Japan); Aminex HPX-87H column (Bio-Rad, Hercules, CA, USA), mobile phase 5 mM sulfuric acid, mobile flow 0.6 mL.min^−1^, injection volume 20 µL, oven temperature 40 °C). Pyruvate was analyzed by a Shimadzu LC-20AT UV‒Vis detector, Bio-Rad Aminex HPX-87H column, 5 mM sulfuric acid mobile phase, 0.8 mL.min^−1^ mobile flow, 20 µL injection volume, and 40 °C oven temperature.

## Results and discussion

### Effects of OLR and HRT on fermentation performance

The effects of different HRT and OLR on VFA production were studied over one year of acidogenic fermentation. Two substrate combinations were used: i) PTMB-VIN mixture (days 1–244) and ii) PTMB-GLY mixture (days 245–346). The concentrations of individual VFA (expressed as VFA-COD) and their corresponding yields are summarized in Table 4.

**Table 4:** Fermented effluent and biogas characteristics from the AR over in the PTMB-VIN phases (I-A, I-B, I-C and I-D) and PTMB-GLY phases (II-A and II-B) (mean ± standard deviation)

Variables	I-A	I-B	I-C	I-D	II-A	II-B
pH	6.3 ± 0.2	5.1 ± 0,3	5.5 ± 0.5	5.3 ± 0.2	5.5 ± 0.2	5.4 ± 0.3
ORP (mV)	−345 ± 12	−251 ± 49	−284 ± 44	−286 ± 32	18 ± 85	−40 ± 34
COD (g.L^−1^)	18.7 ± 4.7	13.4 ± 1.8	35.3 ± 8.4	25.6 ± 3.7	26.8 ± 11.3	36.1 ± 5.3
TS (g.L^−1^)	14.5 ± 0.8	10.2 ± 0.1	22.9 ± 5.3	-	-	21.9 ± 1.9
TVS (g.L^−1^)	9.2 ± 1.1	7.1 ± 0.1	14.9 ± 3.8	-	-	16.9 ± 1.4
NH_4_^+^-N (mg.L^−1^)	14.3 ± 4.2	57.5 ± 38.6	178 ± 102	84.8 ± 48.7	-	-
Biogas (mL.d^−1^)	889 ± 637	647 ± 545	603 ± 234	772 ± 101	33.1 ± 2.5	123 ± 82
YVFA (% w/w)	14.38 ± 1.28	30.1 ± 5.7	39 ± 6.7	25.7 ± 8	1.8 ± 0.6	2.6 ± 1.1
HAc (mg.L^−1^)	219 ± 155	1437 ± 562	4884 ± 1624	4298 ± 3491	278 ± 122	591 ± 265
HProp (mg.L^−1^)	969 ± 385	1102 ± 295	4058 ± 1265	2524 ± 2134	80.4 ± 48.1	321 ± 164
HBut (mg.L^−1^)	1001 ± 841	1254 ± 429	2544 ± 736	3311 ± 3032	86.3 ± 54.7	195 ± 140
HPent (mg.L^−1^)	1519 ± 177	1050 ± 155	2073 ± 309	2135 ± 1860	58.7 ± 28.7	135 ± 119
HCap (mg.L^−1^)	133.3 ± 51.6	157 ± 51	296.6 ± 84.5	462 ± 527	4.7 ± 3.3	16.5 ± 33.6

#### PTMB-VIN mixtures (phases I-A, I-B, I-C, and I-D)

**Fig. 1:**
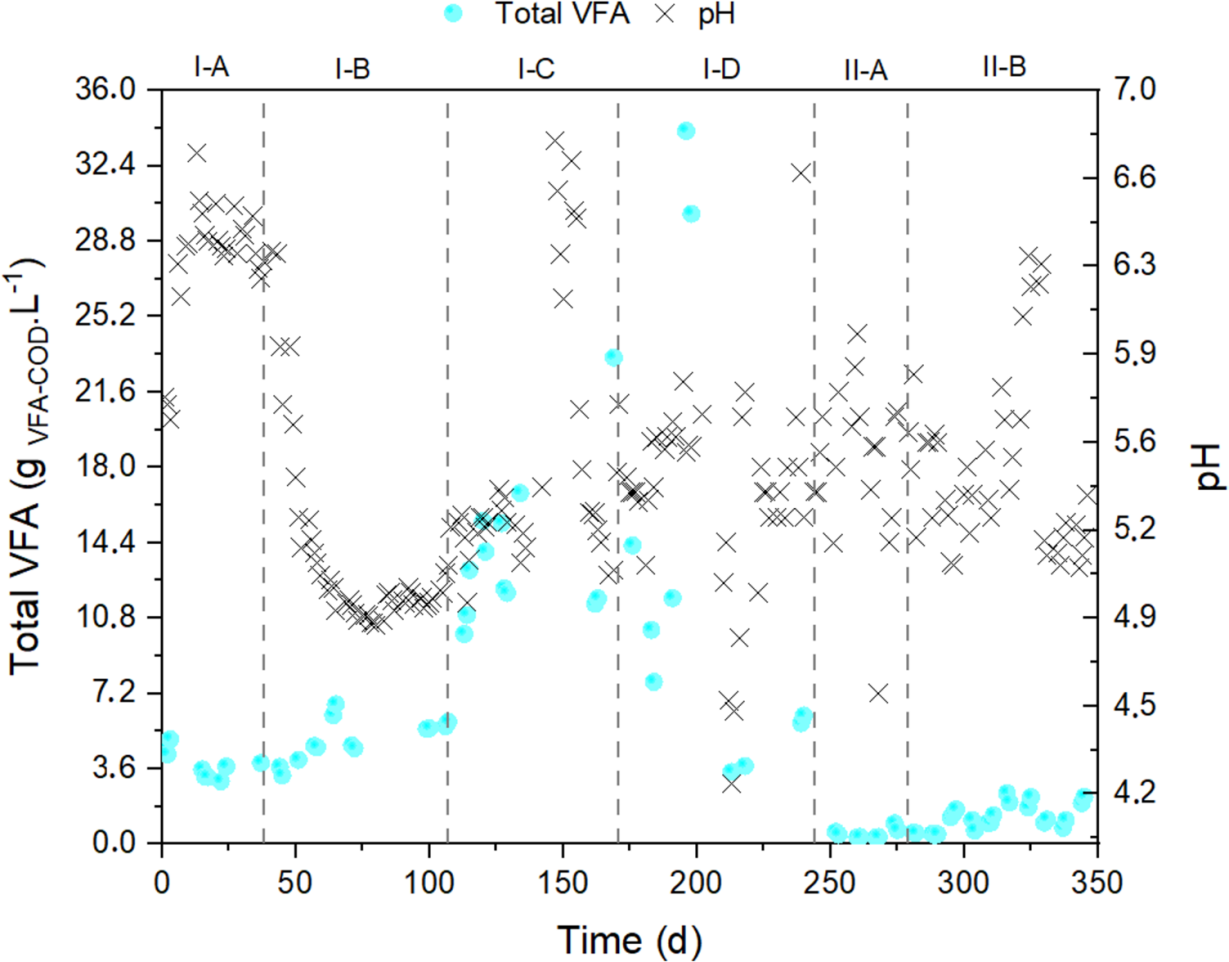
Scatterplot of total VFA production and pH values when tests were performed with PTMB + VIN (phases I-A, I-B, I-C, I-D) and PTMB + GLY (phases II-A and II-B)

The first experimental phase was performed on PTMB-VIN at an HRT of 4 d and an OLR of 5.65 g COD.L^−1^. d^−1^. After the preliminary startup phase, the reactor reached a steady state approximately one week after the beginning of the experimental phase, which corresponded to approximately one HRT. Steady-state conditions were characterized by an average VFA concentration of 3.84 g_VFA_-_COD_. L^−1^, corresponding to a Y_VFA_ of 17.09% w/w (Fig. 1).

A decrease in HRT from 4 to 3 days (phases I-B) on the PTMB-VIN mixture improved VFA productivity. The system achieved VFA concentrations and conversions of 5.0 g_VFA_-_COD_. L^−1^ and Y_VFA_ of 29.66% w/w, respectively, at a 3-day HRT. This excellent performance could be explained by the chemical compounds present in the substrates, specifically VIN, which is usually represented by a very easy chemical structure, such as sugars and alcohols, that require a short time to degrade into VFA (Christofoletti et al. 2013).

A previous study of the DF of sugarcane VIN at mesophilic temperatures revealed a high VFA yield of 33.2% (COD-based) (Eng et al. 2022). This fermentative activity supports the hydrolysis and acidogenic conversion of PTMB during PTMB–VIN co-digestion in the present study. Although co-digestion did not increase the overall VFA yield compared to VIN monodigestion, it benefited from the fermentative capacity of VIN, resulting in comparable conversion efficiencies under continuous and steady-state conditions. Consequently, the use of VIN improved VFA conversion, which is usually lower than that of monofermented MB between 10‒15% w/w (Cho et al. 2015; Silva et al. 2024).

As reported, the 3-d HRT was better than the 4-d HRT owing to enhanced hydrolysis kinetics and VFA conversion into simple compounds. Consequently, an HRT greater than 3 d is associated with a shift from acidogenic fermentation to methanogenesis, with the conversion of VFA into methane (Rizzioli et al. 2024). This is also consistent with the biogas production data obtained from AR. The first experimental phase (I-A), performed at a 4-d HRT, showed the highest biogas production, close to 900 mL.d^−1^ (Table 4).

The 3-d HRT was kept constant in the following experimental phase (I-C), while the OLR was doubled from 5.62 to 11.25 g_COD_.L^−1^d^−1^. Increasing this parameter positively affected acidogenic fermentation, resulting in a VFA concentration of 13.10 g_VFA-COD_. L^−1^ and a conversion yield of 38.53% w/w (Fig. 1). OLR is considered another fundamental parameter in acidogenic fermentation; a low OLR favors the methanogenic phase of anaerobic digestion, whereas high values support the formation of VFA, inhibiting methane bacteria (Bolzonella et al. 2018; Leite et al. 2023). Specifically, different studies have demonstrated that high VFA yields are obtained when the OLR is greater than 10 g_COD_. L^−1^d^−1^ (Strazzera et al. 2021; Silva et al. 2021). However, the OLR was higher than 20 g_COD_. L^−1^d^−1^ can inhibit acidogenic fermentation with unstable VFA production that hinders steady-state conditions (Cheng et al. 2016).

An OLR of 11.25 g_COD_. L^−1^d^−1^ was also tested in the following experimental phase (I-D) but at a lower HRT of 2 d. The results showed similar VFA concentration (12.73 g_VFA-COD_. L^−1^) but a higher Y_VFA_ of 25.7% w/w. Moreover, the outputs were characterized by greater variability in VFA production. This demonstrates that the new HRT was not long enough to ensure optimal and stable hydrolysis and conversion of PTMB-VIN into VFA at 11.25 g_COD_. L^−1^d^−1^ OLR. Thus, the consistent stability observed at 3 d, combined with the reduced performance at 2 d, supports the conclusion that a 3-d HRT represents a suitable and operationally stable condition for PTMB–VIN co-digestion within the tested experimental range.

Although minor variations in COD, C/N ratio, and solid content were inherent to the use of real substrates, the differences in VFA and biogas production observed between phases I-C and I-D were primarily attributed to changes in HRT. At an HRT of 3 d (phases I-C), the anaerobic reactor had higher VFA production, indicating a predominance of acidogenic pathways. When the HRT was reduced to 2 d (phases I-D), total VFA production decreased, while biogas yield slightly increased (Table 4), suggesting conversion of fermentation intermediates into biogas, as mentioned previously. These trends occurred under comparable COD-based organic loading control and steady-state conditions, indicating that the observed changes were driven mainly by HRT reduction rather than by variations in influent COD, C/N ratio, or solid content, which agrees with the established dark fermentation kinetics reported in the literature (Llamas et al. 2022; Pandey et al. 2022).

#### PTMB-GLY mixtures (phases II-A and II-B)

Acidogenic fermentation was also performed on the PTMB-GLY mixture during the last two phases of the experiment, phases II-A and II-B. VFA productivity was low, especially at the lowest HRT of 2 d (0.58 g_VFA_-_COD_. L^−1^) (Fig. 1). -GLY is widely used as a substrate for VFA and methane production by anaerobic digestion. Previous studies have achieved high specific methane production between 300‒380 L_CH4_.kg_VS_^−1^ (Viana et al. 2012). As VFAs are biological intermediates in methane production by anaerobic digestion, the presence of methane indicates the previous formation of these compounds in the same reactor. The low VFA and methane yields obtained from PTMB-GLY fermentation in this study were likely due to the high glycerol content, which inhibited both VFA- and methane-producing microorganisms. Some authors have demonstrated that early-inhibiting effects on metabolic pathways occur when either crude GLY is in the range between 0.9 and 1.5 g.L^−1^ or when the GLY concentration is greater than 1% v/v (Siles López et al. 2009; Fountoulakis et al. 2010). Moreover, from a mechanistic perspective, a fermentation process with a short HRT favors fermentative rather than methanogenic pathways.

Considering the average value of the inlet COD between 30‒35 g.L^−1^ for phases II-A and II-B (Table 4) and the PTMB-GLY ratio of 70:30, it is possible to estimate that the average GLY concentrations found in the inlet mixture were 9.0 and 10.5 gCOD.L^−1^ in phases II-A and II-B, respectively. These values are greater than the inhibition levels reported above and can explain the reduction in the VFA yield. Moreover, the increase in HRT from 2 to 4 d during the last phase (II-B) led to an increase in Y_VFA_ from 2.26% w/w to 3.08% w/w, demonstrating that the system required more time to degrade the intermediates derived from the hydrolysis of GLY.

Although GLY fermentation has been widely reported, including at low HRT (Silva et al. 2024, 2020; Viana et al. 2012; Siles López et al. 2009), complex organic fractions in PTMB likely limited hydrolysis and acidogenic conversion and reduced performance during phase II-A (2-day HRT). The higher Y_VFA_ observed at 4 d (phase II-B) suggests that longer retention times are required to improve VFA recovery in PTMB–GLY co-fermentation, highlighting the relevance of further investigation into HRT and substrate interactions.

### Effect of HRT and OLR on individual VFA dynamics

**Fig. 2:**
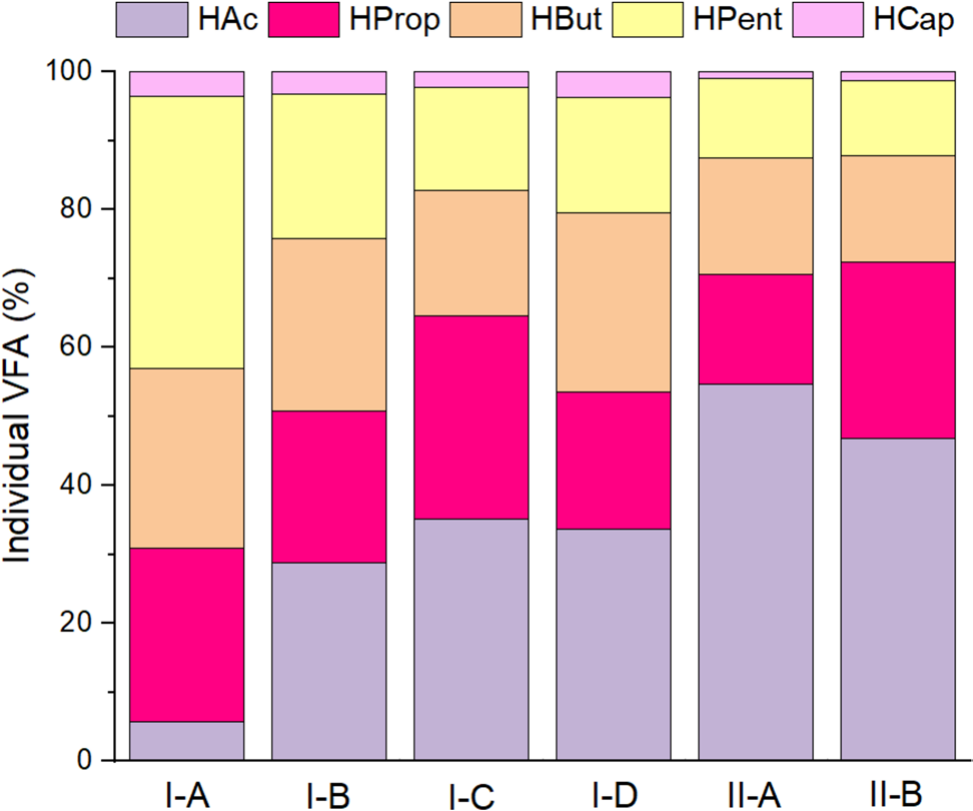
VFA distribution throughout the experimental phases. Abbreviations are given as follows: HAc (acetic), HProp (propionic), HBut (butanoic), HPent (pentanoic), and HCap (caproic). The individual VFA composition weight represents the average VFA production in each phase

Changes in HRT and OLR also influenced the VFA profile. During I-A, 4-d HRT and 5.62 gCOD. L^−1^d^−1^, pentanoic and propionic acids were the major individual VFA, accounting for nearly 40% and 25% (w/w) of the total VFA, respectively (Fig. 2). Increase in OLR to 11.25 gCOD. L^−1^d^−1^ and a reduction in the HRT to 2 d (experimental phase I-D) favored the shortest VFA formation, namely acetic and propionic acids, which accounted for approximately 34% and 20%, respectively, of the total VFA. This finding is consistent with previous studies on acidogenic fermentation, which showed that the production of longer VFA, such as butyric, pentanoic, and caproic acids, usually requires longer HRT and specific molar ratios between electron donor compounds, such as ethanol and lactic acid, and electron acceptors, such as VFA (Cavalcante et al. 2017; Possente et al. 2022).

Regarding the VFA profile, HPent (40%), HBut (21%), and HProp (25%) were predominant in phases I-A (Fig. 2). The dominance of odd-chain acids is associated with the presence of proteins in the substrate, whereas HAc is the primary product (Regueira et al. 2020; Llamas et al. 2022). This phase accounted for the lowest percentage of HAc (6%), which may indicate the presence of acetoclastic archaea consuming HAc. In this strategy, the HProp/HAc ratio exceeded 1.4 (4.4), which is a conventional threshold associated with process failures (Zhang et al. 2014). Considering the aforementioned, DF was conducted at a lower HRT to wash out methanogens in the next phase.

In phase I-B, a new HRT of 3 days and an adjusted pH of 5.5 enhanced the accumulation of VFA compared to the previous phase. The decrease in pH during this phase was verified (Fig. 1). Moreover, the VFA profiles changed, as HAc, HProp, and HBut were the major individual acids at 29, 22%, and 25%, respectively (Fig. 2).

In phase I-C, doubling the OLR (11.25 gCOD. L^−1^d^−1^) under the same HRT (3 d) was analyzed. pH tends to decrease at high OLR (Zhang et al. 2014; Strazzera et al. 2021). In the current study, the pH of the feed influent was corrected to 6.0, resulting in 5.5 ± 0.5 and the highest average VFA yield of the whole experiment (previously discussed). The VFA profiles of phases I-C were as follows: HAc (34%), HProp (30%), and HBut (18%).

As shown in Table 3, phases I-D was being conducted with a lower HRT and the same OLR (2 d and 11.25 g COD. ·L^−1^·d^−1^, respectively). A decrease in the VFA concentration was observed and was attributed to a new VIN batch (collected from the sugarcane ethanol plant), which had lower COD and higher TKN concentrations than the previous VIN feedstock. This also explains the high standard deviations from the mean values listed in Table 1. Thus, at the 2-day HRT, there was a decrease in the COD concentration of the influent feed, which decreased the TKN dilution. Predominant individual VFA were, in descending order: HAc (34%), HProp (30%) and HBut (18%). The C/N ratio of the influent feed was below 10 (Table 2) and the total VFA production was approximately 12 g_VFA-COD_. L^−1^. Zheng et al. (2021) reported that low C/N ratios inhibit AD owing to competition among bacteria, which hinders steady-state conditions.

When VIN was replaced by GLY and the operating conditions of phases I-D were maintained, strategy II-A started, which was the most unfavorable combination for VFA production. This could be related to an overload condition, insufficient retention time for the microorganisms to metabolize the cosubstrates, and/or the presence of unidentified toxic compounds. In both configurations, HAc was the predominant acid, followed by HProp, and HBut (Fig. 2). The higher percentage of HAc may be attributed to an increase in alcohol production upon replacing VIN with GLY. Alcohol fermentation by acetogenic bacteria at mesophilic temperatures leads to HAc production (Quispe et al. 2013; Wang et al. 2020). During cellular energy production, more NAD^+^ is produced via the HProp synthesis pathway rather than the HBut pathway. When there is a high yield of NADH in the medium, HProp forms naturally, such that there is an adequate NADH/NAD^+^ ratio (Silva et al. 2020). The pH showed similar results for both phases (Fig. 1), which was reflected in similar VFA profiles when the proportions of HAc and HProp varied (Fig. 2). The pH was adjusted to 5.0 with 1 M HCl to minimize the formation of 1.3 propanediol, whose optimum pH range is between 6.5 and 7.5 (Asopa et al. 2022).

### Influence of ORP on VFA production

The redox potential was monitored daily throughout the experiment. According to Wang et al. (2020), redox potential can be either managed externally or determined by biochemical reactions, which can support metabolic shifts and the subsequent formation of different metabolites.

#### PTMB-VIN mixtures

As discussed, the chemical compounds present in the substrates, especially in the vinasse used in phases I-A and I-D, require less time to degrade and convert into VFA (Wang et al. 2020).

**Fig. 3:**
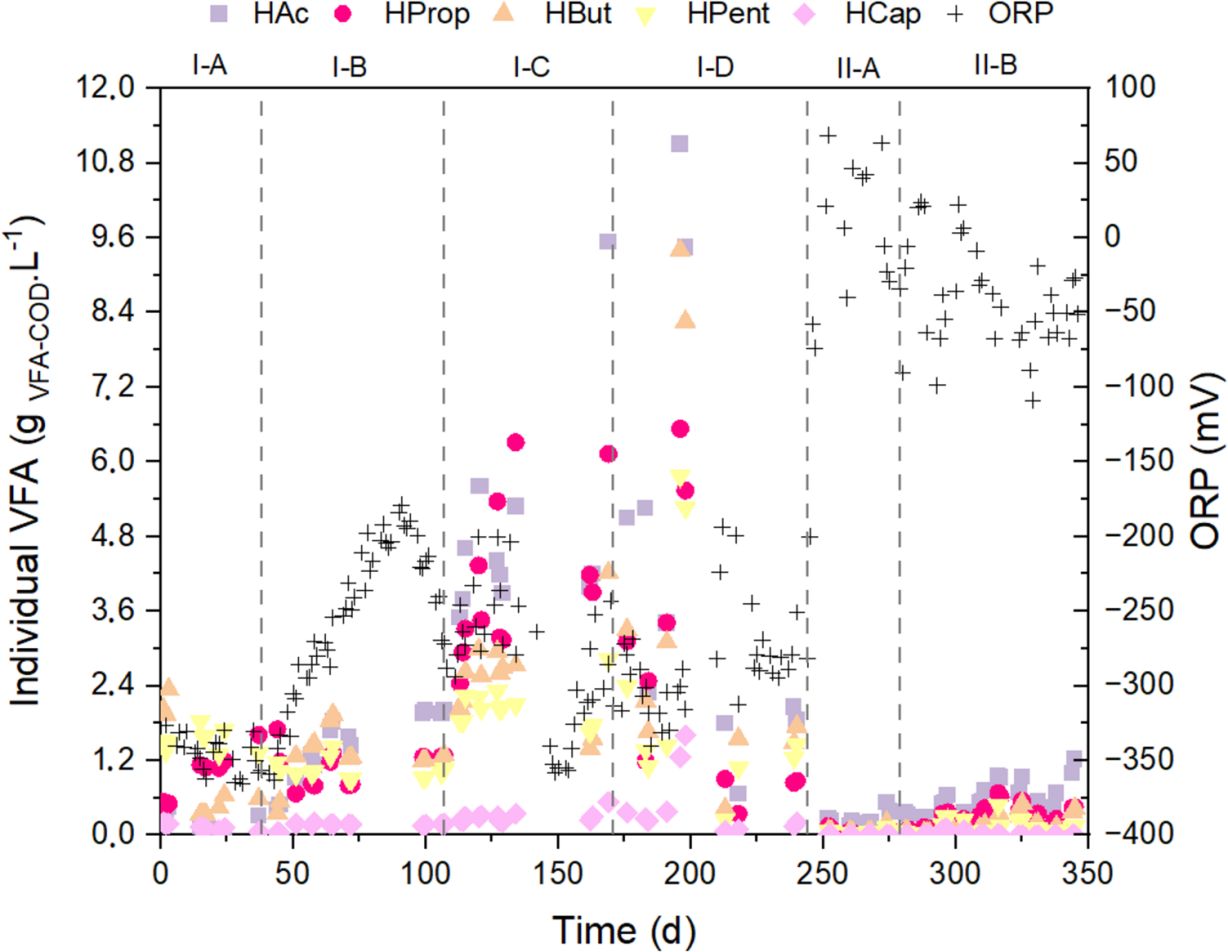
Relationship between the redox potential and the individual VFA concentrations measured across the experiment

In the first experimental phase, it can be concluded that the 4-d HRT promoted anaerobic reactions with an ORP ranging between −300 and −400 mV (Fig. 3, Table 4). According to Yin et al. (2016), a pH below 6.0 and an ORP between −200 and −300 mV may hinder acidogenesis. This is in accordance with Pandey et al. (2022) and Wang et al. (2014), who reported that methanogenesis can only occur below −230 mV.

Reducing the HRT from 4 to 3 d (phase I-B) resulted in the washout of methanogenic organisms from the reactor, supporting an increase in the ORP and reconfiguration of the VFA profile (Fig. 2 and 3). In the other strategies, the ORP remained between −200 and −330 mV (Table 4 and Fig. 3) with an abundance of HAc, HProp, and HBut acids (Fig. 2). Between days 181 and 266, a period that included phases I-D and II-A, the reactor was fed with an open headspace, and during the period when VIN was the co-substrate, the ORP was below −200 mV, that is, there was no significant transfer of oxygen from the air to the liquid medium, and therefore, the redox potential did not vary. Outcomes obtained by Yin et al. (2016) reported a maximum concentration of 29.4 gVFA-COD. L^−1^ when the ORP was kept between −100 and −200 mV whereas a lower VFA yield was obtained when the ORP was varied from −200 to −300 mV. Another study reported that acidogenesis is favored between −100 and −250 mV (Lim et al. 2014). In these studies, butanoic, acetic, and propanoic acids were detected in high proportions. In this study, a maximum concentration of 34 g_VFA-COD_. L^−1^ was reached in phase I-D, as shown in Fig. 1.

#### PTMB-GLY mixture

As seen in phases I-D, the ORP value likely did not change when open headspace feeding was used; however, as soon as glycerol was introduced (phase II-A), there was a 106% increase in the redox potential, resulting in positive ORP values (Table 4 and Fig. 3).

According to Chen et al. (2013) a pure culture took 13 h to fully convert a high glycerol content (60 g.L^−1^) into byproducts including HProp in large amounts at 15 mV redox. Between phases II-A and II-B, there was an increase in the proportion of HProp and a decrease in HAc, which is in line with the results of the above study. However, the co-fermentation in the present study was performed using an open microbiome (mixed biomass). Therefore, HProp accumulation did not have a positive effect on the entire bacterial community, supporting the hypothesis that acidogenic activity is inhibited during phases II-A and II-B.

According to Vesga-Baron et al. (2021), variations in ORP directly affect metabolic processes through the intracellular NADH/NAD^+^ ratios. Consequently, dark fermentation reactions, including ferredoxin and formate hydrogenase reactions, are affected. The authors also emphasized that the main enzymes involved in H_2_ production are affected, and that ORP levels also influence dark fermentation byproducts (metabolites).

Another hypothesis for the low VFA productivity and higher ORP in phases II-A and II-B is the influence of the glycerol salinity. In the present study, the sodium content of glycerol was 31.2 gNa^+^. L^−1^. Therefore, the average Na^+^ concentrations in the reaction medium during phases II-A and II-B were 178.5 and 84.9 mg. L^−1^, respectively, much lower than 3.5 g.L^−1^, the reported limiting Na^+^ concentration to avoid process inhibition (Liu et al. 2017).

### Phase II-B fermentation products

Because of the low VFA yields in phases II-A and II-B, and the increase in ORP, analyses of alcohols, sugars, and pyruvate were performed to specifically identify other DF metabolites of PTMB-GLY.

**Fig. 4:**
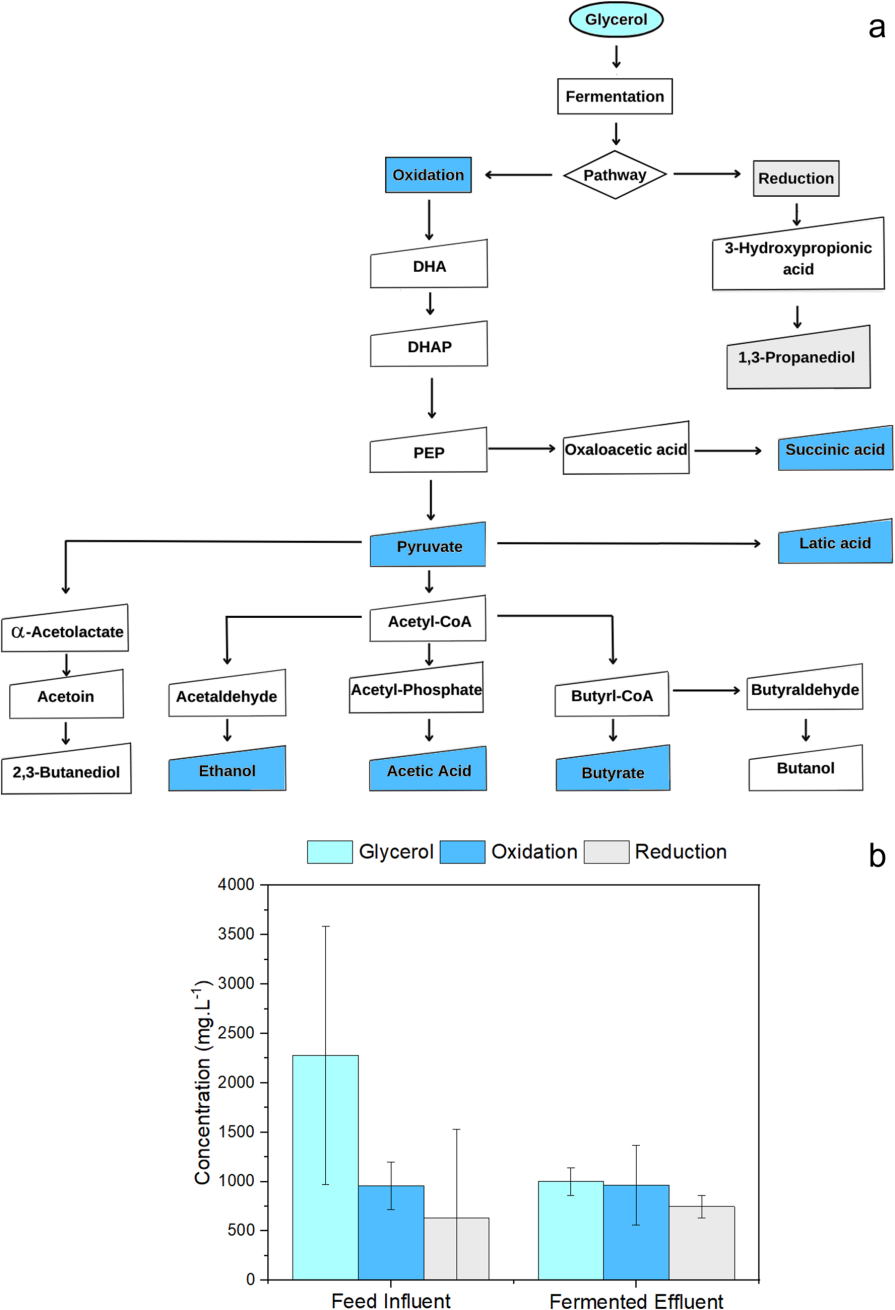
Metabolic pathways for GLY fermentation; highlighted byproducts were analytically determined (adapted from Asopa et al. (2022)) **a**; and composition of metabolites from the oxidative and fermentative pathways **b**. The oxidative pathway was expressed as the sum of HAc, iso-HBut, HBut, HSuc, HLat acids, ethanol and pyruvate. The reductive pathway included 1,3-propanediol

Figure 4a shows the GLY fermentation pathway. This oxidative pathway produces pyruvate, HAc, HBut, iso-HBut, succinic (HSuc) and lactic (HLat) acid, ethanol, and butanol. In contrast, the reductive pathway produces 1,3-propanediol (1,3-PPD). Asopa et al. (2022) and Veras et al. (2020) reported that GLY is reduced in nature and tends to produce reduced byproducts such as 1,3-PPD. It is important to note that no HSuc or 1,3-PPD was detected in the fermented AR 2 (control reactor). Total VFA production in RA2 varied between 0.9 and 3.9 g_VFA-COD_. L^−1^ (Fig. 1) and consisted mainly of HAc, HPro, HBut, and ethanol (200–400 mg_VFA-COD_. L^−1^) (Fig. 2). Thus, the oxidative pathway was favored in the presence of PTMB, whereas glycerol supported reductive metabolism. The results showed that GLY was partially degraded (22%), increasing the oxidative pathway by 12%, which mainly comprises Hac and HBut acids, whereas in the reductive pathway, there was a 10% increase in the concentration of 1,3-PPD (Fig. 4b). Dark fermentation occurred via both metabolic pathways in which VFA competed with the production of 1.3-propanediol.

The coexistence of metabolic pathways is related to the microbiome present in the reactor. Since a mixed bacterial community was used, it can perform both oxidative and fermentative routes, where the transfer of electrons in catabolic and anabolic reactions results in a balanced ORP value (Liu et al. 2013).

### Implications and limitations of PTMB co-fermentation

Using real substrates, such as PTMB, VIN, and GLY, introduced variability in the COD, nutrient content, and solid composition. This variability may influence the fermentation performance. Additionally, the evaluated HRT range was limited (Table 3), and the impact of longer retention times under identical OLR conditions, which could affect the hydrolysis efficiency and VFA recovery, was not assessed. Because methane production and microbial community dynamics have not been directly investigated, the mechanistic interpretation of reductive and oxidative fermentation pathways is limited. Future studies should include parallel PTMB–VIN and PTMB–GLY fermentation under identical operating conditions to better isolate the effects of the different substrates. In addition, lower GLY concentrations should be explored because high GLY levels are likely to impair fermentation performance.

## Conclusion

Acidogenic fermentation at 3 d HRT was associated with higher hydrolysis efficiency and VFA production, whereas longer HRT favored methanogenic activity; high GLY concentration negatively affected the fermentation performance. Increasing the OLR promoted VFA accumulation, reaching up to 34 g VFA-COD L⁻^1^ with conversion efficiencies close to 40%, while also inhibiting methanogenesis. Maximum VFA yields were observed at an ORP of − 310 mV; PTMB–VIN fermentation occurred between − 200 and − 330 mV and favored the production of short-chain VFA (acetate, propionate, and butyrate), whereas GLY fermentation contributed to ORP stabilization near neutral values. Overall, GLY supported reductive metabolic pathways, whereas PTMB favored oxidative routes, indicating that the HRT and OLR exerted distinct effects depending on the substrate composition. These findings provide insight into the fermentation behavior of the PTMB–VIN and PTMB–GLY systems and highlight the key operational factors influencing acidogenic performance.

## Authors´ contributions

The study was originally proposed and designed by Mario Takayuki Kato, Lourdinha Florencio, Wanderli Rogério Moreira Leite. Material preparation, data collection and analysis were performed by Dicla Cesário Pereira de Oliveira, Francyelle Karolynne Vieira Machado, Agnes Adam Duarte Pinheiro. The first draft of the manuscript was written by Dicla Cesário Pereira de Oliveira, Federico Battista, Wanderli Rogério Moreira Leite and all authors commented on previous versions of the manuscript. All authors read and approved the final manuscript.

## Data Availability

The datasets used and/or analysed during the current study are available from the corresponding author on reasonable request.
